# Transoesophageal Echocardiography Related Complications

**Published:** 2009-10

**Authors:** S K Mathur, Pooja Singh

**Affiliations:** 1 Head, Divison of Cardiothoracic Anaesthesia, Anaesthesia, Department of Anaesthesiology, Institute of Medical Sciences,Banaras Hindu University, Varanasi 221005, INDIA; 2 PDCC Student, Division of Cardiothoracic Anaesthesia, Department of Anaesthesiology, Institute of Medical Sciences,Banaras Hindu University, Varanasi 221005, INDIA

**Keywords:** Transesophageal echocardiography, Complications, Prevention

## Abstract

**Summary:**

The application of transesophageal echocardiography (TEE) has been continuously increasing over past several decades. It is usually considered a very safe diagnostic and monitoring device. Though the complications are rare, but these complications must be known to the operators performing TEE. The goal of this article is to encapsulate the potential complications associated with TEE. The complications are primarily related to gastrointestinal, cardiovascular and respiratory systems along with some miscellaneous problems related to probe insertion, drugs and inexperience of the operator. Strategies for the prevention of these complications are also analyzed in order to avoid the risk.

## Introduction

Transesophageal echocardiography (TEE) is a semi invasive monitoring and diagnostic modality of immense utility. The practical clinical use of TEE was first described in 1976 when a modified rigid endoscopic probe with single M-mode crystal was used. Since that time, TEE technology has evolved rapidly with developments in flexible endoscopic probe technology, phased-array ultrasound systems, and crystal miniaturization. Presently, TEE is being used widely in operation theatres, intensive care units, cardiac catheterization laboratory and day care units. Although the technique is quite safe, if conducted by a skilled person, it is important to overview the procedure related complications, considering its widespread use. In the following article, we are trying to give a deep insight regarding the complications of TEE examinations.

## Gastrointestinal Complications:

### 1. Injuries of Gastrointestinal Tract:

Dental trauma^1^, submucosal hematoma of pharyngeal area^2^,^3^ jaw subluxation^4^,^5^ and tonsillar bleeding are related to probe insertion in upper gastrointestinal(GI) tract. Esophageal perforations mostly occur in the abdominal followed by intrathoracic and cervical portions of the esophagus. They are caused by anatomic variations like GI abnormalities, extrinsic compression of esophagus from enlarged left atrium,^6^,^7^ calcified lymph node^8^ and cervical spur. Other causes are poor patient cooperation and inadequate technical skill or mucosal damage due to movement, ischemia, heat and pressure generated by the probe (TEE probe can generate a pressure of 60mmHg ).

The hypopharynx and upper esophagus are most prone to perforation^3^caused by neck extension with or without prominent anterior vertebral osteophytes and by stretching of mucosa and muscular fibres. Shearing forces, prolonged flexion of probe tip and probe mobilization in a locked position can lead to tearing of oesophagus.^9^

Non pulsatile flow, prolonged cardio pulmonary bypass^10^, distended atrium^11^, mechanical compression^12^ and excessive heat are the factors which can cause ischaemic esophageal wall injury.

In conscious and sedated patient, perforations are evident from signs of subcutaneous emphysema, dyspnoea and pain. But under general anaesthesia, esophageal intubation is easy and perforation usually goes unnoticed, ultimately resulting in mediastinitis, sepsis and multi organ failure.^13^ Diagnosis can be confirmed radiologically by computed tomography and chest radiographs and may include findings like pneumothorax, air–fluid level, mediastinal shift, subcutaneous emphysema, pleural effusion and empyema.

### 2. Gastroesophageal Lesions and Anatomic Variations

Lesions such as neoplasm, diverticulum^4^, cervical spine^2^,^14^ or inflammatory mucosal changes are risk factors for complications associated with TEE probe insertion. As there is no direct visualization of esophagus during TEE probe insertion and manipulations, it requires more attention compared to conventional optical gastroscopy. Esophageal intubations most often fail at the level of cricopharynx due to prominence of cricoid muscle. Schatzki's ring and prior cervical surgery^15^ can lead to esophageal narrowing and can cause complications during TEE. Disorders like esophageal achalasia, barrett's esophagus, chemical esophagitis, late scleroderma, Chagas disease and benign and malignant esophageal tumors can reduce esophageal lumen. Peptic ulcer and gastroesophageal reflux disease(GERD) can lead to strictures which ultimately can cause erosion and bleeding of esophagus. Probe of TEE can easily slip into Zenkers diverticulum and can cause perforation.^16^

Normal anatomical variations like aortic impression, large left atrium and left main bronchus or pathological variations such as mediastinal tumours^7^ and esophageal duplication cyst compress esophagus and hamper esophageal intubations.^17^

Vascular abnormalities like esophageal varices due to portal hypertension can cause bleeding during TEE.^18^ Cervical spine abnormalities due to trauma or subluxation at C1 and C2 vertebrae may make esophageal intubation difficult and can also lead to neurological deficit.^19^

### 3. Unsuccessful Esophageal Intubation

Factors contributing to this problem are lack of cooperation from patients and inexperience of operator as well as anatomic abnormalities like double aortic arch^20^, cervical osteophytes^21^ and mucosal abnormalities such as prior radiation exposure, decreased saliva production and prior tracheostomy. Mallory-Weiss syndrome which is associated with forceful vomiting efforts has been reported during TEE which leads to failed intubations.^21^

### 4. Bleeding of Esophageal Tract

Risk factors associated with upper GI bleeding due to TEE include previous ulcerative process, vasoactive drugs and failure to use H2 antagonist drugs in the perioperative period.^22^ Long bypass period, reoperation^23^, emergency surgery, aspirin^24^ and anti-coagulant^25^ use are other factors which are associated with GI bleeding.

### 5. Changes in Esophgeal Integrity

TEE exposes the esophageal mucosa to ultra sound waves and pressure for long periods. Mucosal edema, erosion, hematomas and petechiae can be produced specially in small children.^26^

### 6. Injury to Other Solid Organs & Oral Injuries

Splenic laceration can occur due to deep insertion of the probe into the stomach for transgastric imaging .^27^ Dysphagia can occur due to local compression from probe insertion which affects pharyngoesophageal tissue and laryngeal nerve especially in female and paediatric patients.^28^–^30^ Dysphagia is also associated with pulmonary aspiration. TEE in sitting position can cause dysphasia which is due to local effect of probe, combined with extreme flexion of head.^31^ Tongue swelling^32^ and necrosis^33^ may also occur due to prolonged placement of TEE probe.

### 7. Probe Tip Buckling

Probe tip buckling is caused due to tip flaccidity in an old TEE probe, improper insertion, general anaesthesia and inexperience. It can cause injury when withdrawn hastily.^31^,^34^

### 8. Other Foreign Bodies in Esophagus

Breakage and dislodgement of temperature probe and esophageal stethoscope during TEE are reported.^35^,^36^ Nasogastric tube and feeding tube share the same space and thus may lead to shearing, dislodgement of the spur and poor TEE imaging.

## Respiratory Complications:

TEE examinations in sedated patient may be associated with small reduction of O_2_ saturation. Incidence of oxygen desaturation and aspiration increases with obesity 37 and during emergency procedures.^38^ To avoid this complication, oxygen supplementation is advocated in sedated patient. In awake patients, problems such as bronchospasm, laryngospasm, posterior pharyngeal wall hematoma, supraglottic hematoma and stenosis may occur along with pulmonary edema, atelectasis and airway obstruction.^39^–^44^ TEE probe placement, motion and removal may lead to displacement or accidental extubation of endotracheal tube particularly in children.^45^ Compression of pulmonary tree or endotracheal tube may hamper ventilation. 44–48

## Cardiovascular Complications:

Esophageal intubation can induce vagal and sympathetic reflexes such as hypertension or hypotension, tachy arrhythmias or bradycardia and even myocardial infarction.^49^–^51^ Arrhythmias are manifested as non-sustained ventricular and supra ventricular tachy arrhythmias, atrial fibrillation and 3^rd^ degree heart block.^52^ It can also induce angina and myocardial ischemia. Risk factors like sedation along with fasting, patient on anti-hypertensive drugs and also hypoxemia may precipitate heart failure and fatal arrhythmias.^53^,^54^

Valsalva maneuver associated with retching and coughing leads to increase in intrathoracic, central venous and pulmonary pressures and release is associated with abrupt decrease of systemic pressure. Large intrathoracic pressure and associated hemodynamic changes resulting from retching may cause fatal pulmonary embolisation from right atrium mass, 55,56mitral vegetation and left intracardiac thrombus^57^ resulting in stroke, aortic dissection and cardiac tamponade.^58^

## Infections

Risk of bacteremia is associated with TEE and may lead to morbid infections such as endocarditis. The most common organisms responsible for bacteremia after TEE intubation include α-hemolytic streptococcus, staphylococcus aureus and staphylococcus epidermidis. 59

Use of prophylactic antibiotic therapy during TEE, though controversial, is suggested for patients who are immuno suppressed, have prosthetic valves, cyanotic congenital heart disease, surgically constructed shunts and previous history of endocarditis. 60 Contaminated TEE probe and the lubricating jelly are the sources of infection.^61^,^62^ A properly cleaned probe with glutaral-dehyde can reduce the incidence of post TEE infections.

## Medication Related Complications:

### Sedation:

Sedation improves patients’ tolerance to TEE probe insertion and reduces coughing, vomiting and pain. Benzodiazepines, propofol and short acting narcotics are most commonly used for sedation. Side effects of these drugs like respiratory depression, hypotension, agitation and allergy may occur and must be treated promptly.

### Local Anaesthetic Medication:

Local anaesthetic used systemically to blunt the hemodynamic effects of TEE, for superior laryngeal nerve block and in jelly can cause anaphylactic or overdose reactions. Congenital absence of methemoglobin reductase enzyme and topical local anaesthetics like prilocaine, lidocaine and benzocaine can lead to methemoglobinemia.^63^,^64^,^65^ It can be diagnosed by central cyanosis and low Hb saturation unresponsive to oxygen therapy. Dyspnoea, confusion, dizziness, coma and death may occur.

## Miscellaneous complication:

### • Probe contamination:

Disruption of protective probe sheath can create a lumen between core and sheath which can get filled with fluids and contaminants such as glutaraldehyde and which can be ingested during TEE.^66^

### • Location related complication:

TEE in emergency unit, especially in trauma patients, leads to more complications such as death, respiratory insufficiency, hypotension, emesis, agitation and cardiac dysrrhythmias. These are the patients which present with compromised hemodynamic and respiratory conditions and unstable cervical spine damage. These patients are with full stomach and altered sensorium and thus are at increased risk of aspiration. Therefore, endotracheal intubation is highly recommended in these patients.

### • Effect of ultrasound waves on tissues:

Powerful ultrasound beam can cause vibration of gas filled structures leading to hemorrhage and hemolysis.^67^ It can also produce excessive heat and damage of surrounding tissues. But in TEE, low intensity of 5MHz is used which is devoid of any harmful effects^68^,^69^.

### Relative Contraindications of TEE

History

 Dysphagia

 Odynophagia

 Mediastinal radiation

 Recent upper gastrointestinal surgery

 Recent esophagitis

 Thoracic aortic aneurysm

### Esophageal pathology

 Stricture

 Tumour

 Diverticulum

 Varices

 Esophagitis

### Prevention of TEE complications

Evaluation and surveillance of patients:

1. Informed consent must be obtained.
2. Careful medical history.
  - Allergy.
  - Bleeding disorder.
  - Dysphagia to solid and liquid.
  - Esophageal varices, diverticulum, esophageal web, upper GI bleeding, peptic ulcer, GERD & hiatal hernia.
  - Previous gastric, esophageal and neck surgeries.
  - Radiation therapy.
  - Cervical arthrosis.
  - Use of antacid, salicylates, anticoagulants and antiplatelet agents.
3. Physical Examination.
  - The oral and dental hygiene and loose teeth.
  - Assessment of neck mobility, stability and arthritic changes.
  - Assessment of airway.
4. Endocarditis prophylaxis for high risk patients.
5. Fasting for 6 hr before an elective procedure.
6. Surveillance and monitoring of vital signs at baseline and throughout the procedure.
7. O2 supplementation and venous access should be established. Suction device and resuscitation equipments must be kept ready.
8. In emergency settings, rapid sequence induction with orotracheal intubation is performed while in elective procedures, TEE can be performed on awake or mildly sedated patient with 6 hr fasting.
9. Dentures should be removed and bite guard should be placed to protect instrument and fingers.
10. TEE probe should be lubricated and kept in unlocked control-wheel position. It should never be forced into the passage. TEE probe must be inspected for mechanical dysfunction and damage of outer sheath causing electrical and thermal injuries leading to arrhythmias and death.^70^
11. Awake patient is asked to swallow while under general anesthesia probe can be placed under direct laryngoscopy which reduces the trauma.
12. Insertion of probe only upto 40-50 cm from incisors is advocated. Any nasogastric or feeding tube or temperature probe should be removed to avoid potential, kinking, knotting or gastric migration and prevent interference during imaging.
13. During cardiac surgery special care is taken as the probe is used for longer duration and anticoagulation during cardiopulmonary by-pass and hypothermia leave the mucosa more vulnerable to pressure necrosis and ischemia.
14. Patient should be monitored until fully awake and eating and drinking is allowed once the effect of local anesthetic is dissipated.

Transesophageal echocardiography provides better imaging of cardiac anatomy and function but since it is more invasive than transthoracic echocardiography, operators should be aware of the likely complications , minimize the risk factors and take measures to prevent the complications.

## References

[1] Rafferty T, LaMantia KR, Davis E (1993). Quality assurance for intraoperative transesophageal echocardiography monitoring: a report of 846 procedures. Anesth Analg.

[2] Bettex D, Chassot P D (2002). Échographie Transoesophagienne en Anesthésie-Réanimation.

[3] Saphir JR, Cooper JA, Kerbavez RJ, Larson SF, Schiller NB (1997). Upper airway obstruction after transesophageal echocardiography. J Am Soc Echocardiogr.

[4] Vignon P, Gueret P, Chabernaud JM (1993). Failure and complications of transesophageal echocardiography. Apropos of 1500 consecutive cases (French). Arch Mal Coeur Vaiss.

[5] Chee TS, Quek SS, Ding ZP, Chua SM (1995). Clinical utility, safety, acceptability and complications of transoesophageal echocardiography (TEE) in 901 patients. Singapore Med J.

[6] Massey SR, Pitsis A, Mehta D, Callaway M (2000). Oesophageal perforation following perioperative transoesophageal echocardiography. Br J Anaesth.

[7] Dewhirst WE, Stragand JJ, Fleming BM (1990). Mallory-Weiss tear complicating intraoperative transesophageal echocardiography in a patient undergoing aortic valve replacement. Anesthesiology.

[8] Pong MW, Lin SM, Kao SC, Chu CC, Ting CK, Tsai SK (2003). Unusual cause of esophageal perforation during intraoperative transesophageal echocardiography monitoring for cardiac surgery-a case report. Acta Anaesthesiol Sin.

[9] St-Pierre J, Fortier LP, Couture P, Hebert Y (1998). Massive gastro intestinal hemorrhage after transoesophageal echocardiography probe insertion. Can J Anaesth.

[10] Kharasch ED, Sivarajan M (1996). Gastroesophageal perforation after intraoperative transesophageal echocardiography. Anesthesiology.

[11] Han YY, Cheng YJ, Liao WW, Ko WJ, Tsai SK (2003). Delayed diagnosis of esophageal perforation following intraoperative transesophageal echocardiography during valvular replacement—a case report. Acta Anaesthesiol Sin.

[12] Fujii H, Suehiro S, Shibata T, Aoyama T, Ikuta T (2003). Mallory-weiss tear complicating intraoperative transesophageal echocardiography. Circ J.

[13] oong W, Afifi S, McGee EC (2006). Delayed presentation of gastric perforation after transesophageal echocardiography for cardiac surgery. Anesthesiology.

[14] Tam JW, Burwash IG, Ascah KJ (1997). Feasibility and complications of single-plane and biplane versus multi-plane transesophageal imaging: a review of 2947 consecutive studies. Can J Cardiol.

[15] Dougherty Thomas B, Benumof J (1996). The difficult airway in conventional head and neck surgery. Airway Management Principles and Practice.

[16] Fergus I, Bennett ES, Rogers DM, Siskind S, Messineo FC (2004). Fluoroscopic balloon-guided transesophageal echocardiography in a patient with Zenker's diverticulum. J Am Soc Echocardiogr.

[17] Carerj S, Paola TM, Oddo A, Lucisano V, Oreto G (1998). Esophageal duplication cyst: a rare obstacle to transesophageal echocardiography. Echocardiography.

[18] Suriani RJ, Cutrone A, Feierman D, Konstadt S (1996). Intraoperative transesophageal echocardiography during liver transplantation. J Cardiothorac Vasc Anesth.

[19] Riazi J, Benumof J (1996). The difficult pediatric airway. Airway Management Principles and Practice.

[20] Stevenson JG (1995). Role of intraoperative transesophageal echocardiography during repair of congenital cardiac defects. Acta Paediatr Suppl.

[21] Badaoui R, Choufane S, Riboulot M, Bachelet Y, Ossart M (1994). Esophageal perforation after transesophageal echocardiography (French). Ann Fr Anesth Reanim.

[22] Norton ID, Pokorny CS, Baird DK, Selby WS (1995). Upper gastrointestinal haemorrhage following coronary artery bypass grafting. Aust N Z J Med.

[23] Leitman IM, Paull DE, Barie PS, Isom OW, Shires GT (1987). Intra-abdominal complications of cardiopulmonary bypass operations. Surg Gynecol Obstet.

[24] Kallmeyer IJ, Collard CD, Fox JA, Body SC, Shernan SK (2001). The safety of intraoperative transesophageal echocardiography: a case series of 7200 cardiac surgical patients. Anesth Analg.

[25] Massa N, Morrison M (2003). Transesophageal echocardiography: an unusual case of iatrogenic laryngeal trauma. Otolaryngol Head Neck Surg.

[26] Greene MA, Alexander JA, Knauf DG (1999). Endoscopic evaluation of the esophagus in infants and children immediately following intraoperative use of transesophageal echocardiography. Chest.

[27] Olenchock SA, Lukaszczyk JJ, Reed J (2001). Theman TE. Splenic injury after intraoperative transesophageal echocardiography. Ann Thorac Surg.

[28] Rousou JA, Tighe DA, Garb JL (2000). Risk of dysphagia after transesophageal echocardiography during cardiac operations. Ann Thorac Surg.

[29] Sakai T, Terao Y, Miyata S, Hasuo H, Haseba S, Yano K (1999). Postoperative recurrent laryngeal nerve palsy following a transesophageal echocardiography (Japanese). Masui.

[30] Kohr LM, Dargan M, Hague A (2003). The incidence of dysphagia in pediatric patients after open heart procedures with transesophageal echocardiography. Ann Thorac Surg.

[31] Cucchiara RF, Nugent M, Seward JB, Messick JM (1984). Air embolism in upright neurosurgical patients: detection and localization by two-dimensional transesophageal echocardiography. Anesthesiology.

[32] Yamamoto H, Fujimura N, Namiki A (2001). Swelling of the tongue after intraoperative monitoring by transesophageal echocardiography (Japanese). Masui.

[33] Sriram K, Khorasani A, Mbekeani KE, Patel S (2006). Tongue necrosis and cleft after prolonged transesophageal echocardiography probe placement. Anesthesiology.

[34] Kronzon I, Cziner DG, Katz ES (1992). Buckling of the tip of the transesophageal echocardiography probe: a potentially dangerous technical malfunction. J Am Soc Echocardiogr.

[35] Yasick A, Samra SK (1995). An unusual complication of transesophageal echocardiography. Anesth Analg.

[36] Benedict PE, Foley K (1997). Transesophageal echocardiography not without pitfalls. J Cardiothorac Vasc Anesth.

[37] Dhariwal A, Plevris JN, Lo NT, Finlayson ND, Heading RC, Hayes PC (1992). Age, anemia, and obesity associated oxygen desaturation during upper gastrointestinal endoscopy. Gastrointest Endosc.

[38] Gendreau MA, Triner WR, Bartfield J (1999). Complications of transesophageal echocardiography in the ED. Am J Emerg Med.

[39] Chan KL, Cohen GI, Sochowski RA, Baird MG (1991). Complications of transesophageal echocardiography in ambulatory adult patients: analysis of 1500 consecutive examinations. J Am Soc Echocardiogr.

[40] Khandheria BK, Seward JB, Bailey KR (1991). Safety of transesophageal echocardiography: experience with 2070 consecutive procedures. J Am Coll Cardiol.

[41] Liu JH, Hartnick CJ, Rutter MJ, Hartley BE, Myer CM (2000). 3rd. Subglottic stenosis associated with transesophageal echocardiography. Int J Pediatr Otorhinolaryngol.

[42] Stienlauf S, Witzling M, Herling M, Harpaz D (1998). Unilateral pulmonary edema during transesophageal echocardiography. J Am Soc Echocardiogr.

[43] Lam J, Neirotti RA, Hardjowijono R, Blom-Muilwijk CM, Schuller JL, Visser CA (1997). Transesophageal echocardiography with the use of a four-millimeter probe. J Am Soc Echocardiogr.

[44] Phoon CK, Bhardwaj N (1999). Airway obstruction caused by transesophageal echocardiography in a patient with double aortic arch and truncus arteriosus. J Am Soc Echocardiogr.

[45] Stevenson JG (1999). Incidence of complications in pediatric transesophageal echocardiography: experience in 1650 cases. J Am Soc Echocardiogr.

[46] Gilbert TB, Panico FG, McGill WA, Martin GR, Halley DG, Sell JE (1992). Bronchial obstruction by transesophageal echocardiography probe in a pediatric cardiac patient. Anesth Analg.

[47] Stevenson JG, Sorensen GK (1993). Proper probe size for pediatric transesophageal echocardiography. Am J Cardiol.

[48] Zestos MM, Chehade M, Mossad E (1998). A transesophageal echocardiography probe causes airway obstruction in an older child. J Cardiothorac Vasc Anesth.

[49] Stoddard M F, Longaker RA (1993). The safety o f transesophageal echocardiography in the elderly. Am Heart J.

[50] Al Moussarih A, Douard H, Lafitte S, Broustet JP, Roudaut R (1999). Acute myocardial infarction during transesophageal echocardiography. Echocardiography.

[51] Suriani RJ, Tzou N (1995). Bradycardia during transesophageal echocardiographic probe manipulation. J Cardiothorac Vasc Anesth.

[52] Daniel WG, Erbel R, Kasper W (1991). Safety of transesophageal echocardiography. A multicenter survey of 10,419 examinations. Circulation.

[53] Khandheria BK (1994). The transesophageal echocardiographic examination: is it safe?. Echocardiography.

[54] Goland S, Shimoni S, Attali M (2005). Fatal ventricular arrhythmia as a complication of transesophageal echocardiography. Eur J Echocardiogr.

[55] Shah CP, Thakur RK, Ip JH, Xie B, Guiraudon GM (1996). Management of mobile right atrial thrombi: a therapeutic dilemma. J Card Surg.

[56] Cavero MA, Cristobal C, Gonzalez M, Callego JC, Oteo JF, Artaza M (1998). Fatal pulmonary embolization of a right atrial mass during transesophageal echocardiography. J Am Soc Echocardiogr.

[57] Black IW, Cranney GB, Walsh WF, Brender D (1992). Embolization of a left atrial ball thrombus during transesophageal echocardiography. J Am Soc Echocardiogr.

[58] Kim CM, Yu SC, Hong SJ (1997). Cardiac tamponade during transesophageal echocardiography in the patient of circumferential aortic dissection. J Korean Med Sci.

[59] Rey JR, Axon A, Budzynska A, Kruse A, Nowak A (1998). Guidelines of the European Society of Gastrointestinal Endoscopy (E.S.G.E.) antibiotic prophylaxis for gastrointestinal endoscopy. European Society of Gastrointestinal Endoscopy. Endoscopy.

[60] Wilson W, Taubert KA, Gewitz M (2007). Prevention of infective endocarditis: guidelines from the American Heart Association: a guideline from the American Heart Association Rheumatic Fever, Endocarditis, and Kawasaki Disease Committee, Council on Cardiovascular Disease in the Young, and the Council on Clinical Cardiology, Council on Cardiovascular Surgery and Anesthesia, and the Quality of Care and Outcomes Research Interdisciplinary Working Group. Circulation.

[61] Webb SF (1975). Outbreak of Serratia marcecens associated with flexible fiberbronscope. Chest.

[62] Anonymous (1988). Infection control during gastrointestinal endoscopy. Guidelines for clinical application. Gastrointest Endosc.

[63] Grauer SE, Giraud GD (1996). Toxic methemoglobinemia after topical anesthesia for transesophageal echocardiography. J Am Soc Echocardiogr.

[64] Ho RT, Nanevicz T, Yee R, Figueredo VM (1998). Benzocaine-induced methemoglobinemia—two case reports related to transesophageal echocardiography premedication. Cardiovasc Drugs Ther.

[65] Vidyarthi V, Manda R, Ahmed A, Khosla S, Lubell DL (2003). Severe methemoglobinemia after transesophageal echocardiography. Am J Ther.

[66] Venticinque SG, Kashyap VS, O'Connell RJ (2003). Chemical burn injury secondary to intraoperative transesophageal echocardiography. Anesth Analg.

[67] Baggs R, Penney DP, Cox C (1996). Thresholds for ultrasonically induced lung hemorrhage in neonatal swine. Ultrasound Med Biol.

[68] Carstensen EL, Duck FA, Meltzer RS, Schwarz KQ, Keller B (1992). Bioeffects in echocardiography. Echocardiography.

[69] Miller MW, Brayman AA (1997). Biological effects of ultrasound. The perceived safety of diagnostic ultrasound within the context of ultrasound biophysics: a personal perspective. Echocardiography.

[70] Switz DM, Clarke AM, Longacher JW Jr (1976). Electrical malfunction at endoscopy. Possible cause of arrhythmia and death. JAMA.

